# Supplementary material for: SARS-CoV-2 Nsp6 damages Drosophila heart and mouse cardiomyocytes through MGA/MAX complex-mediated increased glycolysis
Source: Commun Biol. 2022 Sep 30;5:1039. doi: 10.1038/s42003-022-03986-6 (PMC9523645; doi:10.1038/s42003-022-03986-6)
Supplement: Supplementary file 2 — Supplementary Information [file 42003_2022_3986_MOESM2_ESM.pdf]

SUPPLEMENTARY INFORMATION

SARS-CoV-2 Nsp6 damages *Drosophila* heart and mouse cardiomyocytes through MGA/MAX complex-mediated increased glycolysis

Jun-yi Zhu[1,2], Guanglei Wang[3], Xiaohu Huang[1,2], Hangnoh Lee[1,2], Jin-Gu Lee[1,2], Penghua Yang[3], Joyce van de Leemput[1,2], Weiliang Huang[4,5], Maureen A. Kane[4], Peixin Yang[3], Zhe Han[1,2]*

1. Center for Precision Disease Modeling, Department of Medicine, University of Maryland School of Medicine, 670 West Baltimore Street, Baltimore, MD, 21201 USA; 2. Division of Endocrinology, Diabetes and Nutrition, Department of Medicine, University of Maryland School of Medicine, 670 West Baltimore Street, Baltimore, MD, 21201 USA; 3. Department of Obstetrics, Gynecology & Reproductive Sciences, University of Maryland School of Medicine, Baltimore, MD, 21201 USA; 4. Department of Pharmaceutical Sciences, University of Maryland School of Pharmacy, Baltimore, MD, 21201 USA; 5. Present address: University of Queensland, Brisbane, QLD, 4072, Australia; * Corresponding author email: zhan@som.umaryland.edu

Supplementary Fig. 1: Fly progeny from UAS *SARS-CoV-2* crosses for mortality at eclosion assay.

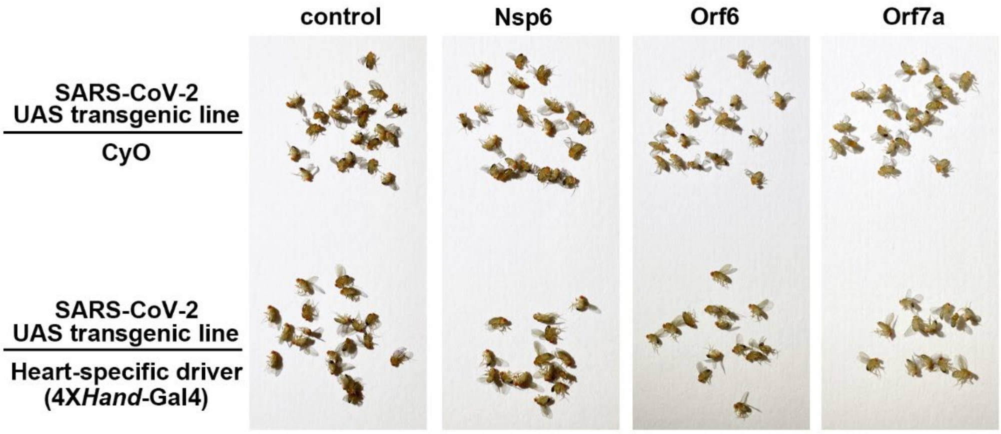

Images of adult progeny emerging from the pupa stage for the crosses in Fig. 1a. Distinct wing phenotypes when carrying the balancer (CyO curly wing; no viral transgene expression) or when expressing the SARS-CoV-2 gene driven by the heart-specific driver 4X*Hand*-Gal4 (straight wing). w^{1118} serves as a wild-type control.

Supplementary Fig. 2: Gene Ontology (GO) term analysis for genes with downregulated expression in the heart of flies expressing SARS-CoV-2 Nsp6.

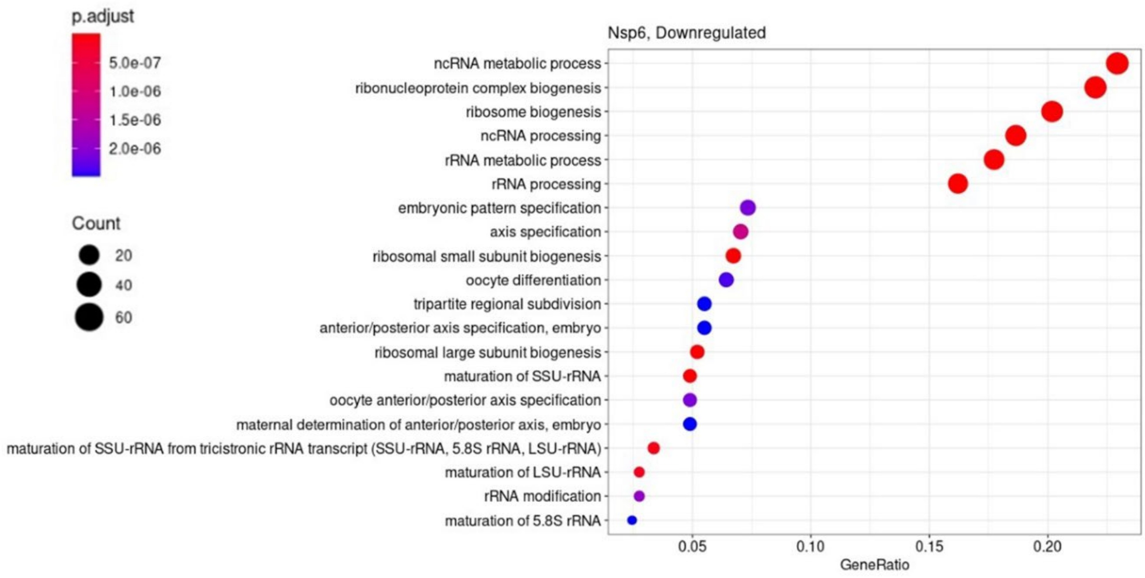

Supplementary Fig. 3: Dosage-lethality curve for 2DG treatment in *Drosophila*.

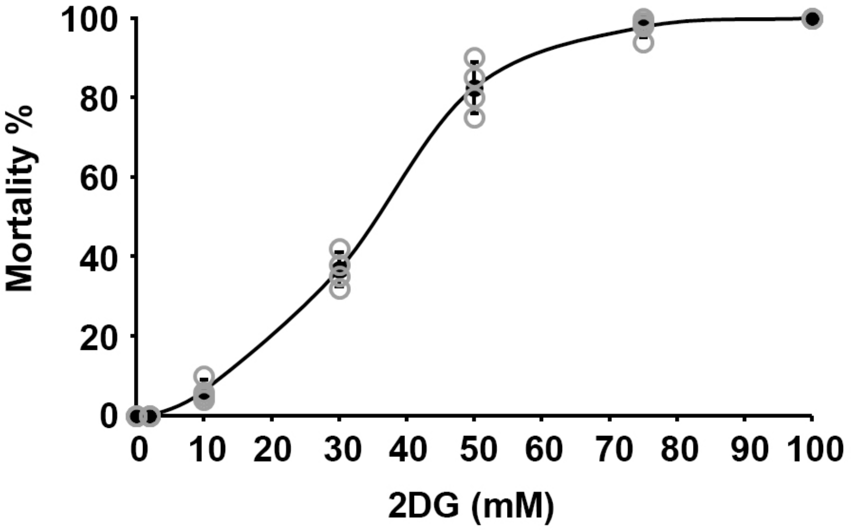

On the X-axis, concentration of 2-deoxy-D-glucose (2DG) in mM; on the Y-axis the percent mortality in wild-type, control flies. Mortality was calculated as (CyO wing – straight wing) / CyO wing × 100. n=4 repeats (~50 flies/vial).

Supplementary Table 1: Primer sequences used for RT-PCR in mouse primary cardiomyocytes.

1. Gpi1 (NM_008155.4)

F: CCATCAAGGTGGACGGCAAAGA

R: CCGTGATGGATTTGCCAGTGTAC

2. Pfkm (NM_001163487.1)

F: CTGTTCGCTCTACCGTGAGGAT

R: TTGGAACCACCTTGACCAGTCC

3. Pgam2 (NM_018870.3)

F: CTGGAATGAGGAGATCGCACCT

R: ATTCCAGTGGGCAGGTTCAGCT

4. Eno1 (NM_001379127.1)

F: TACCGCCACATTGCTGACTTGG

R: GCTTGTTGCCAGCATGAGAACC
