# Supplementary material for: SARS-CoV-2 Nsp6 damages Drosophila heart and mouse cardiomyocytes through MGA/MAX complex-mediated increased glycolysis
Source: Commun Biol. 2022 Sep 30;5:1039. doi: 10.1038/s42003-022-03986-6 (PMC9523645; doi:10.1038/s42003-022-03986-6)
Supplement: Supplementary file 3 — Description of Additional Supplementary Files [file 42003_2022_3986_MOESM3_ESM.pdf]

Description of Additional Supplementary Files

File name: Supplementary Video 1
Description: Representative 15 second video of the beating heart in a wild-type, control fly.

File name: Supplementary Video 2
Description: Representative 15 second video of the beating heart in a fly expressing SARSCoV-2 Nsp6.

File name: Supplementary Data 1
Description: Differentially expressed genes in SARS-CoV-2 Nsp6 expressing fly heart (summary data for RNAseq).

File name: Supplementary Data 2
Description: SARS-CoV-2 Nsp6 interacting proteins in HEK 293T cells identified by mass spectrometry.

File name: Supplementary Data 3
Description: Excel file with source data for the graphs in the manuscript.
