# Supplementary material for: SARS-CoV-2 Nsp6 damages Drosophila heart and mouse cardiomyocytes through MGA/MAX complex-mediated increased glycolysis
Source: Commun Biol. 2022 Sep 30;5:1039. doi: 10.1038/s42003-022-03986-6 (PMC9523645; doi:10.1038/s42003-022-03986-6)
Supplement: Supplementary file 9 — Reporting Summary [file 42003_2022_3986_MOESM9_ESM.pdf]

nature portfolio

Corresponding author(s): Zhe Han

Last updated by author(s): Sep 1, 2022

Reporting Summary

Nature Portfolio wishes to improve the reproducibility of the work that we publish. This form provides structure for consistency and transparency in reporting. For further information on Nature Portfolio policies, see our Editorial Policies and the Editorial Policy Checklist.

Statistics

For all statistical analyses, confirm that the following items are present in the figure legend, table legend, main text, or Methods section.

n/a	Confirmed	
☐	☒	The exact sample size (*n*) for each experimental group/condition, given as a discrete number and unit of measurement
☐	☒	A statement on whether measurements were taken from distinct samples or whether the same sample was measured repeatedly
☐	☒	The statistical test(s) used AND whether they are one- or two-sided<br>*Only common tests should be described solely by name; describe more complex techniques in the Methods section.*
☒	☐	A description of all covariates tested
☐	☒	A description of any assumptions or corrections, such as tests of normality and adjustment for multiple comparisons
☐	☒	A full description of the statistical parameters including central tendency (e.g. means) or other basic estimates (e.g. regression coefficient) AND variation (e.g. standard deviation) or associated estimates of uncertainty (e.g. confidence intervals)
☐	☒	For null hypothesis testing, the test statistic (e.g. *F*, *t*, *r*) with confidence intervals, effect sizes, degrees of freedom and *P* value noted<br>*Give P values as exact values whenever suitable.*
☒	☐	For Bayesian analysis, information on the choice of priors and Markov chain Monte Carlo settings
☒	☐	For hierarchical and complex designs, identification of the appropriate level for tests and full reporting of outcomes
☒	☐	Estimates of effect sizes (e.g. Cohen's *d*, Pearson's *r*), indicating how they were calculated

Our web collection on statistics for biologists contains articles on many of the points above.

Software and code

Policy information about availability of computer code

Data collection	Classification of glycolysis, pentose phosphate pathway (PPP), citric acid (a.k.a. TCA) cycle, and oxidative phosphorylation (OxPhos) were based on KEGG database annotations (Kanehisa et al., 2016). The gene conservation scores (fly-human) were obtained from the DRSC Integrative Ortholog Prediction Tool (DIOPT) version 8 (Hu et al., 2011). | Zeiss ZEN (Blue edition 3.0 flies/3.4 mice) was used for image acquisition. Thorlange OCT (version 5.2.1.0) was used for cardiac function acquisition. SparkControl software (version 2.3) was used for assays on the microplate reader.
Data analysis	The short-reads, generated from the RNAseq, were mapped to the Berkeley Drosophila Genome Project (BDGP) reference genome release 6 (BDGP 6) using HISAT2 version 2.0.5 (Kim et al., 2019) using parameters: --dta --phred33. For gene-level quantification, the Drosophila gene annotation model from Ensembl version 100 (Yates et al., 2020), which corresponds to Flybase 6.28 (Larkin et al., 2021), was used with featureCounts version 1.5.0-p3 (with default parameters). DESeq2 version 1.2.0 was used for differential expression analysis (Love, Huber and Anders, 2014). ClusterProfiler version 3.18.1 (Yu et al., 2012) was used for the Gene Ontology (GO) and KEGG pathway analyses. | ImageJ software (version 1.49) was used for image processing. PAST software (version 4.10) was used for statistical analyses of assay data.

For manuscripts utilizing custom algorithms or software that are central to the research but not yet described in published literature, software must be made available to editors and reviewers. We strongly encourage code deposition in a community repository (e.g. GitHub). See the Nature Portfolio guidelines for submitting code & software for further information.

Data

Policy information about availability of data

All manuscripts must include a data availability statement. This statement should provide the following information, where applicable:

- Accession codes, unique identifiers, or web links for publicly available datasets
- A description of any restrictions on data availability
- For clinical datasets or third party data, please ensure that the statement adheres to our policy

The datasets generated and analyzed during this study have been deposited at public servers. The RNAseq data can be accessed through the NCBI Gene Expression Omnibus (GEO) with the following accession number: GSE173835. The mass spectrometry proteomics data have been deposited to the ProteomeXchange Consortium via the PRIDE partner repository with the dataset identifier PXD036447 | All data supporting the findings of this study are available from the corresponding author on reasonable request.

Human research participants

Policy information about studies involving human research participants and Sex and Gender in Research.

Reporting on sex and gender	n/a
Population characteristics	n/a
Recruitment	n/a
Ethics oversight	n/a

Note that full information on the approval of the study protocol must also be provided in the manuscript.

Field-specific reporting

Please select the one below that is the best fit for your research. If you are not sure, read the appropriate sections before making your selection.

☒ Life sciences ☐ Behavioural & social sciences ☐ Ecological, evolutionary & environmental sciences

For a reference copy of the document with all sections, see nature.com/documents/nr-reporting-summary-flat.pdf

Life sciences study design

All studies must disclose on these points even when the disclosure is negative.

Sample size	No sample-size calculation was performed to predetermine group sizes. Based on prior experience sample size and replicates were determined, these were in line with peer-reviewed publications by us and other groups; including, multiple independent fly lines.
Data exclusions	n/a
Replication	Replicates are indicated in the methods. All replicates reproduced the presented findings. Biological replicates are presented as means with standard deviation. No replicates were conducted for survival and mortality at eclosion assays, which included 100 and 200 flies per group, respectively.
Randomization	Female/male flies were distributed at random over experimental groups. For fly heart morphology imaging and OCT female flies were used. For the adult survival assay male flies were used. | Mice were distributed at random over experimental groups.
Blinding	The investigators were not blinded to group allocation.

Reporting for specific materials, systems and methods

We require information from authors about some types of materials, experimental systems and methods used in many studies. Here, indicate whether each material, system or method listed is relevant to your study. If you are not sure if a list item applies to your research, read the appropriate section before selecting a response.

Materials & experimental systems

n/a	Involved in the study	
☐	☒	Antibodies
☐	☒	Eukaryotic cell lines
☒	☐	Palaeontology and archaeology
☐	☒	Animals and other organisms
☒	☐	Clinical data
☒	☐	Dual use research of concern

Methods

n/a	Involved in the study	
☒	☐	ChIP-seq
☒	☐	Flow cytometry
☒	☐	MRI-based neuroimaging

Antibodies

Antibodies used	The following stain was used for imaging: Alexa Fluor 647 Phalloidin (Thermo Fisher; 1:1,000) | The following antibodies were used for immunostaining: Mouse anti-ATP5A antibody (Abcam, ab14748; 1:1,000); Goat anti-mouse IgG (H+L) highly cross-adsorbed secondary, Alexa Fluor 488 (Thermo Fisher; 1:1,000)
Validation	Phalloidin, Alexa Fluor 647 (Thermo Fisher): Selectively stains F-actin, Excitation/emission = 650/668 nm, For fixed and permeabilized samples; Certificate of Analysis specification used by manufacturer : Absorption max. 653 +/- 3nm; Fluorescence emission max. 673 +/- 4nm; HPLC purity >= 90% at 647nm (https://www.thermofisher.com/order/catalog/product/A22287). | Mouse monoclonal ATP5a (Abcam, ab14748) was validated by the manufacturer (https://www.abcam.com/atp5a-antibody-15h4c4-mitochondrial-marker-ab14748.html); Purity, IgG fraction (IgG2b; kappa light chain), near homogeneity as judged by SDS-PAGE - the antibody was produced in vitro using hybridomas grown in serum-free medium, and then purified by biochemical fractionation; Clone number, 15H4C4; Suitable for ICC-IF, and more; Reacts with Drosophila melanogaster and more. | Goat anti-mouse IgG (H+L), Alexa Fluor 488 (Thermo Fisher) was validated by the manufacturer (https://www.thermofisher.com/antibody/product/Goat-anti-Mouse-IgG-H-L-Highly-Cross-Adsorbed-Secondary-Antibody-Polyclonal/A-11029).

Eukaryotic cell lines

Policy information about cell lines and Sex and Gender in Research

Cell line source(s)	HEK 293T: human embryonic kidney cell line, purchased from ATCC
Authentication	HEK 293T was authenticated by ATCC
Mycoplasma contamination	Confirmed that HEK 293T tested negative for mycoplasma contamination.
Commonly misidentified lines (See ICLAC register)	HEK 293T are not lised in ICLAC

Animals and other research organisms

Policy information about studies involving animals; ARRIVE guidelines recommended for reporting animal research, and Sex and Gender in Research

Laboratory animals	Drosophila melanogaster (generated previously (Zhu et al., 2021); purchased from Bloomington Drosophila Stock Center) | Wild-type C57BL/6J mice (purchased from Jackson Laboratory; #000664)
Wild animals	The flies and mice are inbred strains.
Reporting on sex	For fly heart morphology imaging and OCT female flies were used. For the adult survival assay male flies were used. | For both female and male mice were used.
Field-collected samples	No samples/animals were collected in the field.
Ethics oversight	Fly studies not considered animal research by IACUC. | For mice, the procedures for animal use were approved by the University of Maryland School of Medicine Institutional Animal Care and Use Committee (IACUC).

Note that full information on the approval of the study protocol must also be provided in the manuscript.
